# Oncologic impact of delay between diagnosis and radical nephroureterectomy

**DOI:** 10.3389/fonc.2022.1025668

**Published:** 2022-12-14

**Authors:** Kuan-Hsien Wu, Chao-Hsiang Chang, Hsi-Chin Wu, Steven K. Huang, Chien-Liang Liu, Cheng-Kuang Yang, Jian-Ri Li, Jen-Shu Tseng, Wun-Rong Lin, Chih-Chin Yu, Chi-Wen Lo, Chao-Yuan Huang, Chung-Hsin Chen, Chung-You Tsai, Pai-Yu Cheng, Yuan-Hong Jiang, Yu-Khun Lee, Yung-Tai Chen, Ting-Chun Yeh, Jen-Tai Lin, Yao-Chou Tsai, Thomas Y. Hsueh, Bing-Juin Chiang, Yi-De Chiang, Wei-Yu Lin, Yeong-Chin Jou, See-Tong Pang, Hung-Lung Ke

**Affiliations:** ^1^ Department of Urology, Kaohsiung Medical University Hospital, Kaohsiung, Taiwan; ^2^ Department of Urology, China Medical University and Hospital, Taichung, Taiwan; ^3^ School of Medicine, China Medical University, Taichung, Taiwan; ^4^ Department of Urology, China Medical University Beigang Hospital, Yunlin, Taiwan; ^5^ Division of Urology, Department of Surgery, Chi Mei Medical Center, Tainan, Taiwan; ^6^ Department of Medical Science Industries, College of Health Sciences, Chang Jung Christian University, Tainan, Taiwan; ^7^ School of medicine, Kaohsiung Medical University, Kaohsiung, Taiwan; ^8^ Division of Urology, Department of Surgery, Taichung Veterans General Hospital, Taichung, Taiwan; ^9^ Institute of Medicine, Chung Shan Medical University, Taichung, Taiwan; ^10^ Department of Urology, MacKay Memorial Hospital, Taipei, Taiwan; ^11^ Department of Medicine, Mackay Medical College, Taipei, Taiwan; ^12^ Institute of Biomedical Informatics, National Yang Ming Chiao Tung University, Taipei, Taiwan; ^13^ Division of Urology, Department of surgery, Taipei Tzuchi Hospital, The Buddhist Tzu Chi Medical Foundation, New Taipei City, Taiwan; ^14^ School of Medicine, Buddhist Tzu Chi University, Hualien, Taiwan; ^15^ Department of Urology, National Taiwan University Hospital, College of Medicine, National Taiwan University, Taipei, Taiwan; ^16^ Divisions of Urology, Department of Surgery, Far Eastern Memorial Hospital, New Taipei City, Taiwan; ^17^ Department of Electrical Engineering, Yuan Ze University, Taoyuan, Taiwan; ^18^ Department of Biomedical Engineering, National Taiwan University, Taipei, Taiwan; ^19^ Department of Urology, Hualien Tzu Chi Hospital, Buddhist Tzu Chi Medical Foundation and Tzu Chi University, Hualien, Taiwan; ^20^ Department of Urology, Taiwan Adventist Hospital, Taipei, Taiwan; ^21^ Division of Urology, Department of Surgery, Kaohsiung Veterans General Hospital, Kaohsiung, Taiwan; ^22^ Department of surgery, Taipei Tzu chi Hospital, The Buddhist Tzu Chi Medical Foundation, New Taipei City, Taiwan; ^23^ Department of Urology, Taipei Medical University Hospital, Taipei Medical University, Taipei, Taiwan; ^24^ Division of Urology, Department of Surgery, Taipei City Hospital Renai Branch, Taipei, Taiwan; ^25^ Department of Urology, School of Medicine, National Yang Ming Chiao Tung University, Taipei, Taiwan; ^26^ College of Medicine, Fu-Jen Catholic University, New Taipei City, Taiwan; ^27^ Department of Urology, Cardinal Tien Hospital, New Taipei City, Taiwan; ^28^ Department of Life Science, College of Science, National Taiwan Normal University, Taipei, Taiwan; ^29^ Department of Urology, School of Medicine, College of Medicine, Taipei Medical University, Taipei, Taiwan; ^30^ Department of Urology, Shuang Ho Hospital, Taipei Medical University, New Taipei City, Taiwan; ^31^ TMU Research Center of Urology and Kidney (TMU-RCUK), Taipei Medical University, Taipei, Taiwan; ^32^ Division of Urology, Department of Surgery, Chang Gung Memorial Hospital at Chiayi, Chiayi, Taiwan; ^33^ Chang Gung University of Science and Technology, Chiayi, Taiwan; ^34^ Department of Medicine, College of Medicine, Chang Gung University, Taoyuan, Taiwan; ^35^ Department of Urology, Ditmanson Medical Foundation Chiayi Christian Hospital, Chiayi, Taiwan; ^36^ Department of Health and Nutrition Biotechnology, Asian University, Taichung, Taiwan; ^37^ Division of Urology, Department of Surgery, Chang Gung Memorial Hospital, Linkou Branch, Taoyuan, Taiwan; ^38^ Department of Urology, School of Medicine, College of Medicine, Kaohsiung Medical University, Kaohsiung, Taiwan; ^39^ Graduate Institute of Medicine, College of Medicine, Kaohsiung Medical University, Kaohsiung, Taiwan; ^40^ Department of Urology, Kaohsiung Municipal Ta-Tung Hospital, Kaohsiung, Taiwan

**Keywords:** urinary tract urothelial carcinoma, surgical wait time, nephroureterectomy, ureteroscopy, survival

## Abstract

**Purpose:**

This study aimed to evaluate the oncological outcome of delayed surgical wait time from the diagnosis of upper tract urothelial carcinoma (UTUC) to radical nephroureterectomy (RNU).

**Methods:**

In this multicenter retrospective study, medical records were collected between 1988 and 2021 from 18 participating Taiwanese hospitals under the Taiwan UTUC Collaboration Group. Patients were dichotomized into the early (≤90 days) and late (>90 days) surgical wait-time groups. Overall survival, disease-free survival, and bladder recurrence-free survival were calculated using the Kaplan–Meier method and multivariate Cox regression analysis. Multivariate analysis was performed using stepwise linear regression.

**Results:**

Of the 1251 patients, 1181 (94.4%) were classifed into the early surgical wait-time group and 70 (5.6%) into the late surgical wait-time group. The median surgical wait time was 21 days, and the median follow-up was 59.5 months. Our study showed delay-time more than 90 days appeared to be associated with worse overall survival (hazard ratio [HR] 1.974, 95% confidence interval [CI] 1.166−3.343, *p* = 0.011), and disease-free survival (HR 1.997, 95% CI 1.137−3.507, *p* = 0.016). This remained as an independent prognostic factor after other confounding factors were adjusted. Age, ECOG performance status, Charlson Comorbidity Index (CCI), surgical margin, tumor location and adjuvant systemic therapy were independent prognostic factors for overall survival. Tumor location and adjuvant systemic therapy were also independent prognostic factors for disease-free survival.

**Conclusions:**

For patients with UTUC undergoing RNU, the surgical wait time should be minimized to less than 90 days. Prolonged delay times may be associated with poor overall and disease-free survival.

## 1 Introduction

Upper tract urothelial carcinoma (UTUC) is a rare malignant tumor, which accounts for 5–10% of all urothelial carcinomas, with an estimated annual incidence of 1–2 cases per 100,000 in Western countries (1). However, this can vary between different geographical regions, age, occupation, and other factors (2). In Taiwan, according to the 2018 Cancer Registry Annual Report published by the Health Promotion Administration Ministry of Health and Welfare, a high incidence of UTUC was discovered, which represented 43% of UCs, especially in the southwest coast region (3).

Radical nephroureterectomy (RNU) with bladder cuff resection is the standard treatment for UTUC (4). Prior to surgery, besides high diagnostic accuracy of computed tomography (CT), ureteroscopy (URS) is still regarded an important step in the diagnosis of UTUC (5). Hence, a patient with localized UTUC receives at least two surgeries (URS and RNU) during the clinical management (6). Compared with bladder cancer, UTUC shows more aggressive nature, and over 60% of patients have invasive disease at the time of diagnosis (7). Urologists recommend that it is necessary for patients with a definite diagnosis to receive surgery immediately (8).

Inevitably, there are variables, such as preoperative evaluation, the pursuit of additional therapeutic opinions, limitations of surgical schedules, and patient-related reasons, that may lead to a delay from symptom onset to diagnosis, and later to surgical treatment (9). Also, the outbreak of coronavirus disease-19 (COVID-19) has had a profound global impact on all aspects of urology health care, in which non-emergency operations were postponed or cancelled. Generally, surgeons believe that prolonged surgical wait time (SWT) may have a negative impact on the patient’s clinical outcome because of the invasiveness of UTUC (8). Some studies have shown that a delay of >3months in treatment for bladder cancer may cause worse survival (10, 11). However, there are still conflicting reports on UTUC. In this study, we evaluated the impact of the delay from diagnosis to radical nephroureterectomy (RNU) on the oncological outcomes of UTUC.

## 2 Methods

### 2.1 Patient population

**Figure 1:**
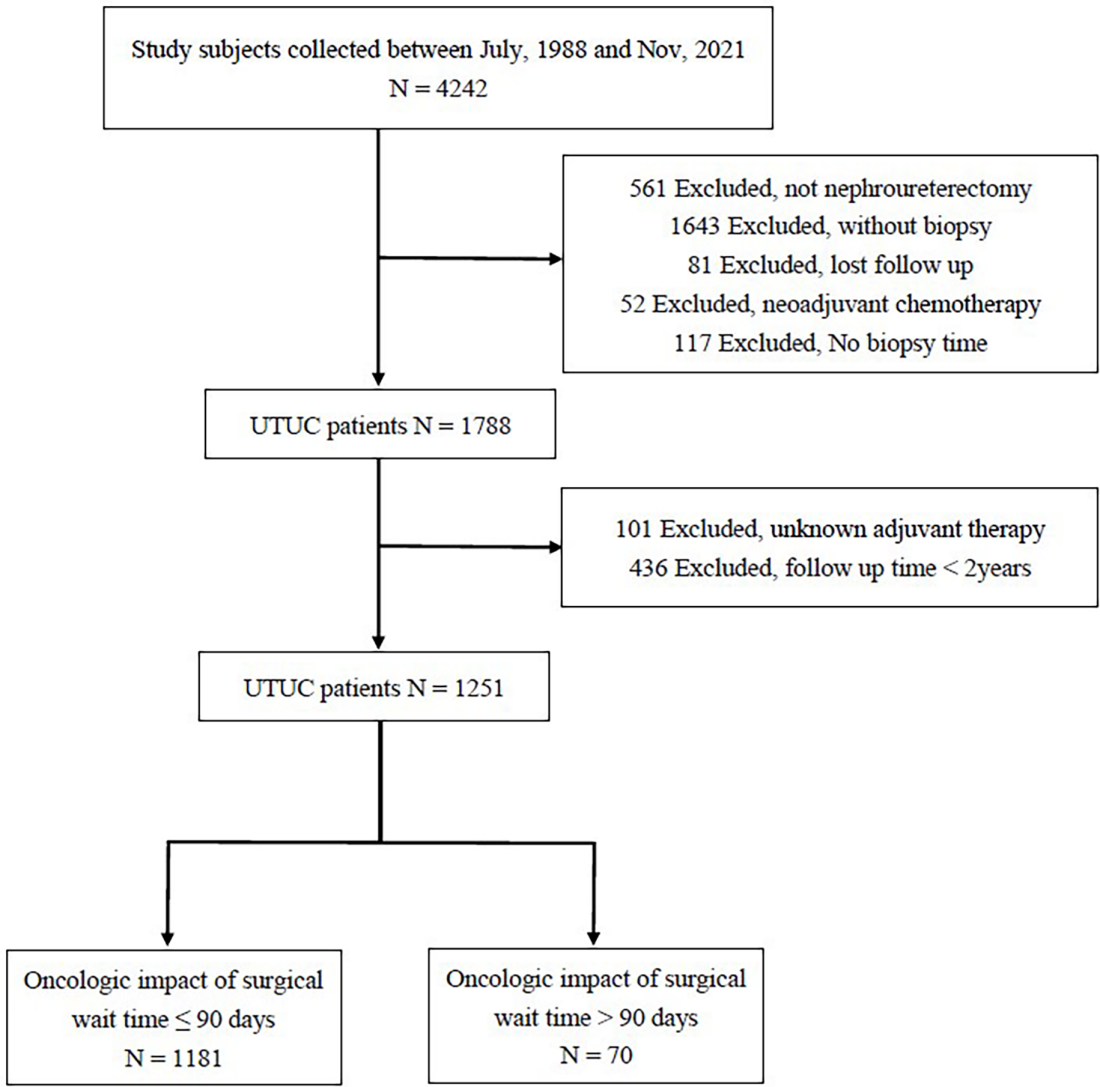
Patient flow diagram for UTUC diagnosis.

This study was approved by our institutional review board [KMUHIRB-E(I)-20180214] and meet the guidelines of the responsible governmental agency. We retrospectively reviewed the medical records of 18 participating Taiwanese hospitals under the Taiwan UTUC Collaboration Group and identified 4242 UTUC patients. The following patients were excluded from the analysis (**Figure 1**): those without nephroureterectomy (n = 561), without biopsy (n = 1643), without biopsy time (n = 117), without regular follow-up (n = 81), and treated with neoadjuvant chemotherapy (n = 52). In addition, we excluded patients with unknown adjuvant therapy (n = 101) and a follow-up time of <2 years (n = 436). We finally included 1251 patients who underwent RNU between July 1988 and November 2021. The patient biopsy dates were from February 14, 2000, to March 2, 2021. The patients’ operation periods were from February 22, 2000, to March 23, 2021. According to different SWTs, the patients were divided into early and late groups.

Various parameters were collected for analysis, including sex, age, ECOG performance status, Charlson Comorbidity Index (CCI), cell type, tumor location, tumor size, and important pathological features such as pathological T stage, pathological N stage, adjuvant systemic therapy, lymphovascular invasion (LVI), surgical margin, and preoperative hydronephrosis.

### 2.2 Statistical methods

Differences between the groups were compared using the two-sample Pearson chi-square test for categorical variables. Continuous variables were tested for normality using the Kolmogorov–Smirnov test. The Kaplan–Meier estimator was used to estimate the rates of prognostic outcomes, and survival curves were compared using the stratified log-rank test. The Cox proportional hazard model was used to assess the effect of the surgical approach on the prognostic outcomes, with and without adjusting for potential confounders. This study used stepwise regression, a method of fitting regression models in which the predictive variables were chosen using an automatic procedure. This study analyzed multiple factors affecting follow-up time and adjuvant use using stepwise linear regression. All relevant covariates, significant and non-significant, in the univariate analysis were included in the variable list to be selected. The significance levels for entry and stay were set to 0.05 and 0.1. The best regression model was then identified manually by reducing the significance levels to 0.05, corresponding to the chosen level. All statistical assessments were two-tailed and considered statistically significant at *p <*0.05. Statistical analyses were performed using IBM SPSS statistical software version 26.

## 3 Results

### 3.1 Population characteristics

A total 1251 patients with UTUC who underwent RNU were enrolled in the study, including 519 men (41.5%) and 732 women (58.5%). The median SWT was 21 days [interquartile range (IQR): 11.00–32.0]. The median follow-up duration was 59.5 months [IQR: 39.3–87.7]. The median age was 67.4 years [IQR: 60.6–75.1].

Clinicopathological characteristics showing the association of time from diagnosis to RNU are shown in **Table 1**. Our study cohort were then classified according to the time from the date of URS biopsy to the date of RNU (≤90 days vs. >90 days), which was defined as SWT. There were 1181 (94.4%) patients in early SWT group and 70 (5.6%) patients in late SWT group. There were no significant differences between the two groups in terms of age, ECOG performance status, CCI, tumor pathological type, tumor location, tumor size, pathological T-stage, pathological N-stage, adjuvant systemic therapy, LVI, surgical margin, preoperative hydronephrosis, mortality, disease-free status, bladder UC after RNU, or follow-up time, except for sex (*p* = 0.025). Men could delay surgery more easily than women.

**Table 1:** Baseline demographic data of variables the UTUC patients.

Variables	≤ 90 days (N = 1181)		> 90 days (N = 70)		*p*-value ^a^
	N	%	N	%
Gender					
Men	481	(40.7)	38	(54.3)	0.025*
Women	700	(59.4)	32	(45.8)	
Age					
<70	658	(56.1)	36	(51.5)	0.454
≥70	517	(44.0)	34	(48.6)	
ECOG performance status
0	423	(44.8)	34	(59.6)	0.142
1	452	(47.9)	18	(31.6)	
2	58	(6.1)	5	(8.8)	
CCI
0	285	(24.1)	18	(25.7)	0.764
≥1	896	(75.9)	52	(74.3)	
Cell Type
Urothelial	1101	(93.2)	69	(98.6)	0.369
Squamous	3	(0.3)	0	(0.0)	
UC with variants	71	(6.0)	1	(1.4)	
Others	6	(0.5)	0	(0.0)	
Tumor location					
Renal pelvis	519	(44.1)	37	(52.9)	0.077
Ureter	444	(37.8)	17	(24.4)	
Renal pelvis + Ureter	215	(18.4)	16	(23.0)	
Tumor size
non-visible	51	(4.3)	5	(7.1)	0.638
<1cm	111	(9.4)	8	(11.4)	
≥1 & < 2 cm	292	(24.8)	14	(20.0)	
≥2 & < 3 cm	273	(23.2)	14	(20.0)	
≥ 3cm	449	(38.3)	29	(41.5)	
Pathological stage T
pTis	19	(1.6)	3	(4.3)	0.370
pTa	194	(16.9)	16	(23.3)	
pT1	352	(30.4)	20	(29.0)	
pT2	265	(22.9)	12	(17.4)	
pT3	314	(27.3)	17	(24.7)	
pT4	12	(1.0)	1	(1.4)	
Pathological stage N
pN0	273	(23.5)	18	(26.1)	0.968
pN1	16	(1.4)	1	(1.4)	
pN2	19	(1.6)	1	(1.4)	
pNx	854	(73.5)	49	(71.0)	
Adjuvant systemic therapy
No	979	(82.9)	59	(84.3)	0.764
Yes	202	(17.2)	11	(15.8)	
Lymphovascular invasion
No	991	(84.7)	55	(78.6)	0.170
Yes	179	(15.4)	15	(21.5)	
Surgical margin
Free	1136	(98.0)	67	(97.2)	0.642
Positive	24	(2.1)	2	(2.9)	
Preoperative hydronephrosis
No	482	(41.0)	29	(41.4)	0.946
Yes	693	(59.1)	41	(58.7)	
Mortality
No	734	(62.2)	41	(58.6)	0.716
UTUC related	98	(8.3)	5	(7.1)	
non-UTUC related	162	(13.7)	13	(18.6)	
Unknown	186	(15.9)	11	(15.8)	
Disease free
No	187	(15.9)	14	(20.1)	0.352
Yes	927	(78.6)	50	(71.5)	
Unknown	67	(5.7)	6	(8.6)	
Bladder UC after RNU
No	714	(60.9)	42	(60.9)	0.330
Yes	375	(32.0)	19	(27.5)	
not available	83	(7.1)	8	(11.6)	
Follow up (months) ^b^ median	59.8	54.5			0.717

ECOG, Eastern Cooperative Oncology Group; CCI, Charlson Comorbidity Index

a Chi-Squared test calculated for the difference Variables.

b Wilcoxon rank-sum test calculated for the difference in medians.

*< 0.05, ** < 0.01

At the time of surgery, 621 patients (49.6%) had muscle-invasive (MI) disease (T2); however, no significant differences were noted between the two groups.

### 3.2 Survival

#### 3.2.1 Overall survival

In total, 633 patients died during the follow-up period. The 5-year OS rates were 84% in the early SWT group and 79% in the late SWT groups, with no significant difference (*p* = 0.145).

Univariate analyses showed that age (*p* < 0.001), ECOG performance status (2, *p* < 0.001), CCI (*p* < 0.001), tumor location (ureter, *p* = 0.030), preoperative hydronephrosis (*p* = 0.012), LVI (*p* < 0.001), surgical margin (*p* < 0.001), and pathological T stage (*p* < 0.001) were associated with poor OS (**Table 2**). Multivariate analysis revealed that SWT (*p* = 0.011), age (*p* = 0.001), ECOG performance status (1 and 2, *p* = 0.048 and *p* < 0.001, respectively), CCI (*p* = 0.032), tumor location (ureter tumor, *p* = 0.022), surgical margin (*p* < 0.001), pathological T-stage (pT2, pT3 and pT4, *p* = 0.043, *p* < 0.001 and *p* = 0.001, respectively), and adjuvant systemic therapy (*p* = 0.030) were independent predictors of OS (**Table 3**).

**Table 2:** Comparative univariate survival analysis the UTUC patients.

Univariate analysis	OS		DFS		BRFS		
	HR (95% CI)	*p*-value	HR (95% CI)	*p*-value	HR (95% CI)	*p*-value	
Group							
≤ 90 days	1		1		1		
> 90 days	1.425 (0.883, 2.298)	0.147	1.555 (0.903, 2.678)	0.112	0.920 (0.580, 1.459)	0.724	
Sex							
Male	1		1		1		
Female	0.926 (0.730, 1.175)	0.527	0.891 (0.674, 1.178)	0.419	0.488 (0.400, 0.596)	<0.001**	
Age							
<70	1		1		1		
≥70	1.915 (1.510, 2.428)	<0.001**	1.368 (1.035, 1.809)	0.028*	0.929 (0.760, 1.137)	0.475	
ECOG performance status							
0	1		1		1		
1	1.336 (0.993, 1.797)	0.056	1.258 (0.917, 1.725)	0.155	1.124 (0.885, 1.426)	0.339	
2	3.641 (2.427, 5.462)	<0.001**	1.797 (1.071, 3.016)	0.026*	1.259 (0.815, 1.947)	0.300	
CCI							
0	1		1		1		
≥1	1.722 (1.270, 2.335)	<0.001**	1.074 (0.776, 1.486)	0.666	0.845 (0.676, 1.055)	0.137	
Tumor size							
<1cm	1		1		1		
≥1 & < 2 cm	0.927 (0.615, 1.398)	0.717	0.765 (0.450, 1.299)	0.321	0.806 (0.587, 1.109)	0.185	
≥2 & < 3 cm	1.095 (0.733, 1.637)	0.657	1.405 (0.869, 2.270)	0.166	0.900 (0.656, 1.233)	0.511	
≥ 3cm	1.132 (0.780, 1.643)	0.514	1.396 (0.889, 2.193)	0.147	0.744 (0.553, 1.002)	0.052	
Tumor location							
Renal pelvis	1		1		1		
ureter	1.337 (1.029, 1.737)	0.030*	1.442 (1.053, 1.976)	0.023*	1.048 (0.837, 1.312)	0.684	
Renal pelvis + ureter	1.261 (0.903, 1.760)	0.174	1.627 (1.121, 2.363)	0.010*	1.346 (1.037, 1.748)	0.025*	
Preoperative hydronephrosis						
No	1		1		1	
Yes	1.393 (1.076, 1.802)	0.012*	1.332 (0.992, 1.789)	0.057	0.871 (0.713, 1.065)	0.177
Lymphovascular invasion						
No	1		1		1	
Yes	1.766 (1.318, 2.368)	<0.001**	2.207 (1.607, 3.032)	<0.001**	1.025 (0.780, 1.348)	0.858
Surgical margin						
No	1		1		1	
Yes	3.235 (1.810, 5.781)	<0.001**	3.241 (1.759, 5.970)	<0.001**	0.640 (0.265, 1.548)	0.322
Pathological stage T						
pTis/pTa/pT1	1		1		1	
pT2	1.261 (0.928, 1.715)	0.139	2.204 (1.507, 3.224)	<0.001**	0.908 (0.700, 1.178)	0.467
pT3	1.672 (1.263, 2.213)	<0.001**	3.256 (2.319, 4.571)	<0.001**	1.063 (0.841, 1.344)	0.607
pT4	5.401 (2.511, 11.618)	<0.001**	6.200 (2.477, 15.518)	<0.001**	0.000 (0.000),	0.926
Pathological stage N						
pN0	1		1		1	
pN+	1.238 (0.588, 2.604)	0.574	2.389 (1.235, 4.622)	0.010*	0.627 (0.290, 1.355)	0.235
pNx	1.025 (0.757, 1.388)	0.872	0.964 (0.689, 1.349)	0.831	1.100 (0.863, 1.403)	0.441
Cell Type						
UC	1		1		1	
Not UC	1.051 (0.652, 1.695)	0.839	1.043 (0.606, 1.796)	0.879	0.624 (0.389, 1.002)	0.051
Adjuvant systemic therapy						
No	1		1		1	
Yes	0.764 (0.534, 1.095)	0.142	1.082 (0.754, 1.553)	0.668	0.778 (0.588, 1.031)	0.081

Cl, confidence; HR, hazard ratio; OS, overall survival; DFS, disease-free survival; BRFS, Bladder Recurrence-free survival; ECOG, Eastern Cooperative Oncology Group; CCI, Charlson Comorbidity Index

* < 0.05, ** < 0.01

**Table 3:** Comparative multivariable survival analysis the UTUC patients.

Multivariable analysis	OS		DFS		BRFS	
	HR (95% CI)	*p*-value	HR (95% CI)	*p*-value	HR (95% CI)	*p*-value
Group						
≤ 90 days	1		1		1	
> 90 days	1.974 (1.166, 3.343)	0.011*	1.997 (1.137, 3.507)	0.016*	0.848 (0.534, 1.346)	0.484
Sex						
Male	1		1		1	
Female	0.762 (0.576, 1.009)	0.058	0.886 (0.653, 1.203)	0.438	0.485 (0.395, 0.595)	<0.001**
Age						
<70	1		1			
≥70	1.662 (1.244, 2.221)	0.001	1.252 (0.940, 1.667)	0.124		
ECOG performance status						
0	1					
1	1.362 (1.003, 1.849)	0.048*	1.244 (0.902, 1.717)	0.184		
2	2.969 (1.918, 4.594)	<0.001**	1.589 (0.938, 2.691)	0.085		
CCI						
0	1					
≥1	1.560 (1.038, 2.344)	0.032*				
Tumor location						
Renal pelvis	1		1			
Ureter	1.454 (1.056, 2.002)	0.022*	1.415 (0.994, 2.013)	0.054		
Renal pelvis + ureter	1.258 (0.851, 1.859)	0.250	1.514 (1.016, 2.257)	0.041*		
Surgical margin						
No	1					
Yes	3.493 (1.791, 6.811)	<0.001**				
Pathological stage T						
pTis/pTa/pT1	1		1			
pT2	1.443 (1.012, 2.056)	0.043*	2.249 (1.484, 3.410)	<0.001**		
pT3	2.173 (1.523, 3.102)	<0.001**	4.430 (2.995, 6.551)	<0.001**		
pT4	4.768 (1.859, 12.231)	0.001**	6.240 (2.211, 17.607)	0.001**		
Adjuvant systemic therapy						
No	1		1			
Yes	0.613 (0.394, 0.953)	0.030*	0.497 (0.325, 0.759)	0.001**		
Cell Type						
UC					1	
Not UC					0.724 (0.544, 0.963)	0.026*

Cl, confidence; HR, hazard ratio; OS, overall survival; DFS, disease-free survival; BRFS, Bladder Recurrence-free survival; ECOG, Eastern Cooperative Oncology Group; CCI, Charlson Comorbidity Index

*< 0.05, ** < 0.01

#### 3.2.2 Disease-free survival

DFS for patients who had RNU ≤90 days and >90 days after diagnosis were 83% and 79% at 5 years after the surgery, respectively. Univariate analyses showed that age (*p* = 0.028), ECOG performance status (2, *p* = 0.026), tumor location (ureter and ureter + renal pelvis, *p* = 0.023 and *p* = 0.010, respectively), LVI (*p* < 0.001), surgical margin (*p* < 0.001), and pathological T stage (*p* < 0.001) were associated with poor DFS. Multivariate analysis revealed that SWT (*p* = 0.016), tumor location (renal pelvic and ureter tumor, *p* = 0.041), pathological T stage (pT2, pT3 and pT4, *p* < 0.001, *p* < 0.001 and *p* = 0.001, respectively), and adjuvant systemic therapy (*p* = 0.001) were independent predictors of DFS.

#### 3.2.3 Bladder recurrence free survival

In univariate analyses, sex (*p* < 0.001) and tumor location (renal pelvic and ureter tumor, *p* = 0.025) were associated with poor BRFS. Multivariate analysis revealed that sex (*p* < 0.001) and pathological tumor type (*p* = 0.026) were independent predictors of BRFS.

#### 3.2.4 Kaplan–Meier analysis

**Figure 2:**
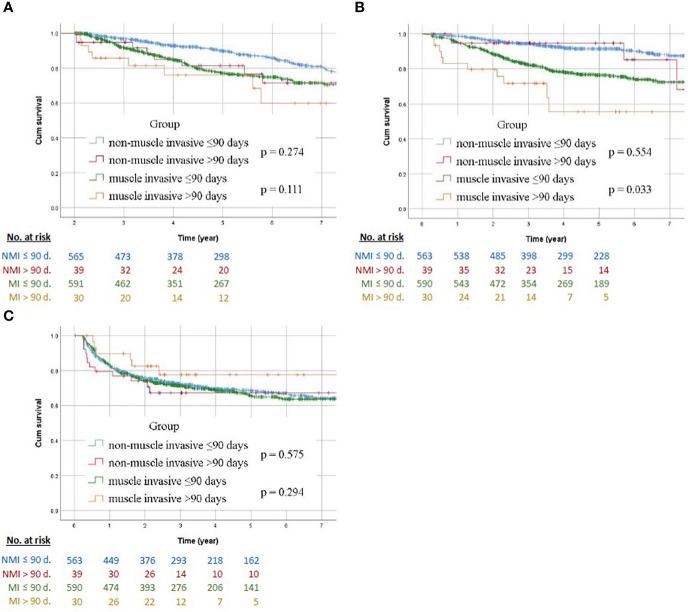
Survival Kaplan–Meier curves in patients with non-muscle invasive and muscle invasive UTUC comparing early and late surgical wait time to nephroureterectomy **(A)** overall survival, **(B)** disease-free survival, and **(C)** bladder recurrence-free survival. NMI, non-muscle invasive; MI, muscle invasive; d., day.

The Kaplan–Meier curve of non-muscle invasive and muscle invasive groups with different surgical wait times are compared in **Figure 2**. Regarding low stage tumor, Kaplan–Meier analysis showed no statistical intergroup differences for OS, DFS (*p* = 0.274, 0.554, respectively). Muscle invasive tumors also revealed no statistical differences in OS between the early and late SWT groups (*p* = 0.111), whereas the early SWT group had a better DFS than the late SWT group (*p* = 0.033). There was no difference between the two groups with respect to BRFS, regardless of the stage.

## 4 Discussion

In Asia, especially in Taiwan, UTUC has a higher prevalence than that in Western countries. In this multicenter retrospective study of a population-based Taiwanese database, the SWT no more than 90 days between diagnosis and radical nephroureterectomy for the UTUC is associated with a better OS and DFS. It is helpful for urologists to recognize the related risk factors. The major carcinogenic factors are occupational exposure to chemical agents and tobacco consumption. Aristolochic acid exposure (Chinese herb nephropathy) is a specific risk factor as well (12). Preoperative (such as tobacco consumption, tumor location, and multi-focality, American Society of Anesthesiology score) and postoperative factors (such as tumor stage and grade, lymph node involvement, and LVI) have been acknowledged to be prognostic factors of UTUC. Once a UTUC lesion is suspected and diagnosed, surgery should be arranged as soon as possible following the guidelines. To our best knowledge, this is the first multicenter large-scale study in Asia to evaluate the oncologic impact of SWT for the UTUC population.

In a previous study, extended SWT beyond a particular threshold has an adverse impact on the patient’s quality of life and psychological health, and even worse clinical outcomes between different urological neoplasms (8). There are some possible factors for the prolonged SWT. Objective factors, such as preoperative evaluation, limitations of the health care system, and seeking second opinions, can contribute to the delay of the surgery, while disease-related factors, may also cause prolonged interval between diagnosis and surgery. In addition, current COVID-19 pandemic has led to significant delay in urological surgeries as well (13). Many large volume hospitals are busy dealing with the pandemic. Lee et al. reported that despite the serious situation of COVID-19, we should still try to avoid delaying the operation (6). Hence, it is important to clarify how SWT affects the oncologic impact and prognosis of UTUC (14).

There is still no consensus regarding SWT for UTUC in previously published studies. This may be due to different inclusion criteria and research methods. Most studies set the cutoff time to 3 months based on previous experience with bladder cancer. Decreased OS and disease-specific survival were observed when the interval between diagnosis and cystectomy was more than 3 months (11). In a literature review, six studies were found to report on the oncologic impact of SWT on UTUC patients. Among studies using 3 months as the cutoff time, Lee et al. and Zhao et al. found worse OS and CSS after a 3-month delay in the RNU group (6, 15). However, some studies have shown contradictory results. Waldert et al. and Sundi et al. showed no significant effect of SWT on CSS and recurrence-free survival (RFS) (9, 16). Furthermore, other time intervals have also been discussed in previous publications. Lee et al. included 138 patients with a cutoff time of 1 month. The results showed that worse CSS and RFS were related to greater SWT in ureter tumor subgroup rather than overall UTUC patients (17). Xia et al. divided the cohort into 6 surgical wait-time groups, from less than 7 days to between 120 and 180 days. A surgical wait time of >120 days was correlated with worse OS (18). In the present study, a delay time of more than 90 days appeared to be associated with worse OS and DFS, and this remained an independent prognostic factor after adjusting for other confounding factors.

The possible reason for no consensus for SWT may be related to different inclusion time among the studies mentioned above. Some involved patients’ first presentation to the outpatient department, whereas others recruited patients with initial CT imaging or URS biopsy. Hematuria may be treated with conservative treatment at first, and further surveys will be conducted. CT is a useful diagnostic tool for UTUC and can be used to detect the detailed anatomy of the urinary tract. It can be used to visualize tumors of the distal ureter and renal pelvis, but calyceal tumors could sometimes be missed according to a previous comprehensive analysis (19). CT scan was correlated to final histopathology with a sensitivity of 89% and an overall accuracy of 74% in 148 patients. URS had similar sensitivity but significantly greater specificity and accuracy when compared with CT (20). Even though diagnostic ureteroscopy seems to increase the time to RNU, previous study showed no statistical differences in CSS, RFS and metastasis-free survival (21). Hence, URS still plays an important role in the diagnosis of UTUC.

In line with previous studies, there was a significant correlation between biopsy grade and surgical tumor grade, and high grade was strongly associated with invasive tumor stage (pT2–T4) (22, 23). In the present study, we found that higher pathological stage T was associated with poor OS and DFS. In addition, the survival curve revealed that a higher tumor stage leads to poor survival outcomes. Our cohort included low-grade and high-grade patients on biopsy. Hence, we analyzed subgroup for the patients with high-grade disease on biopsy. The results revealed SWT more than 90 days appeared to be associated with worse OS (HR 2.147, 95% CI 1.164−3.959, *p* = 0.014), and DFS (HR 2.445, 95% CI 1.259−4.748, *p* = 0.008) in multivariable survival analysis. This remained as an independent prognostic factor after other confounding factors were adjusted. The result was corresponded to all UTUC patients in our study. Hence, if biopsy results indicate a high-grade tumor, the surgery plan should not be delayed because of the association with invasive tumor stage. We strongly recommend that patients with higher grade undergo surgery as soon as possible. Besides, the undergrading and understaging rates were 32% and 46%, respectively (23), so we should not underestimate the low-grade tumor as well and manage the disease within the threshold of 90 days.

Our analysis also showed that age, ECOG performance status, CCI, surgical margin, tumor location and adjuvant systemic therapy were independent prognostic factors for overall survival. Tumor location and adjuvant systemic therapy were independent prognostic factors for disease-free survival. In a subgroup analysis of ureteral urothelial carcinoma by Lee et al., there was a statistically significant difference in CSS and RFS of 1-month SWT to surgery. In accordance with the literature, ureteral location seems to be an independent factor for worse CSS and RFS compared to the renal pelvis (24). The rich blood vessels and lymphatics in the surrounding layer of the ureter may lead to distant metastasis.

In our analysis, no difference was observed in BRFS between the ≤90 and >90 days groups, while sex and cell type were the independent prognostic factors. Bladder recurrence tended to occur more in men, which is consistent with previous studies (25, 26). The meta-analysis has identified significant predictors, such as patient-, tumor-, and treatment-specific factors, of bladder recurrence after RNU. We should also be aware of the possibility of metachronous bladder tumor, and patients should be urged for regular follow-up.

Our study has several limitations. First, it was of retrospective design with some inherent limitations. There was no information on the reasons for the delay in surgical time. Second, there were definite losses during the time of data collection due to the long follow-up time. Moreover, despite the large number of cases, multiple institutions across two decades were involved in this study. Inclusion of diverse backgrounds and surgeons with various levels of experience and possible lack of generalizability of their work due to potentially endemic etiology of UTUC in Taiwan were inevitable, causing potential introduction of bias.

In conclusion, for patients with UTUC undergoing RNU, the SWT should be minimized to less than 90 days. Prolonged wait time may be associated with poor OS and DFS. Further research is required to corroborate our results.

## Data availability statement

The original contributions presented in the study are included in the article/supplementary material. Further inquiries can be directed to the corresponding author.

## Author contributions

H-LK, Y-CT contributed to conception and design of the study. Y-CT, K-HW performed the statistical analysis. K-HW wrote draft of the manuscript. All authors contributed to the article and approved the submitted version.
